# NMN supplementation as a strategy to improve oocyte quality: a systematic review and transcriptomic analysis

**DOI:** 10.1007/s10815-025-03720-1

**Published:** 2025-10-29

**Authors:** Hyunseo Noh, Sioban Sen Gupta, Srividya Seshadri, Xavier Viñals Gonzalez

**Affiliations:** 1 Preimplantation Genetics Group, Institute for Women’s Health, University College London, Bloomsbury, 84-86, Chenies Mews, London, WC1E 6HU UK; 2 Embryology Department, Aria Fertility, 8 Welbeck Way, London, W1G 9YL UK

**Keywords:** Nicotinamide mononucleotide (NMN), Oocyte quality, Mitochondrial function, Oxidative stress, Fertility, Transcriptomics

## Abstract

**Purpose:**

Oocyte quality declines with age and metabolic stress, largely due to mitochondrial dysfunction and NAD⁺ depletion. Nicotinamide mononucleotide (NMN), a precursor of NAD⁺, has emerged as a potential intervention to restore cellular energy metabolism. This study systematically reviews preclinical evidence on NMN supplementation and integrates transcriptomic analysis of human oocytes to assess its relevance in human fertility.

**Methods:**

A systematic review was conducted following PRISMA guidelines across Medline, Embase, and Scopus (January 2015–October 2024). Seven high-quality original studies were included after screening and bias assessment. Data were synthesised through thematic analysis and pathway annotation. Additionally, single-oocyte RNA sequencing was performed on 46 human oocytes at germinal vesicle, metaphase I, and metaphase II stages to profile NAD⁺-related gene expression.

**Results:**

Across animal models, NMN supplementation has been shown to improve mitochondrial regulation, reduce oxidative stress, and modulate apoptotic and inflammatory pathways in response to metabolic, environmental, and ageing stress. Transcriptomic analysis identified 900 differentially expressed genes between germinal vesicle and metaphase II oocytes, with significant changes in mitochondrial and oxidative stress–related genes (i.e. SIRT3, DNM1L, SOD1), aligned with NMN’s known mechanisms of action.

**Conclusions:**

NMN supplementation shows improvements for oocyte function across diverse preclinical models. Human transcriptomic data further highlight mitochondrial and oxidative pathways as key regulatory points during oocyte maturation. Standardised protocols and clinical trials are needed to evaluate NMN’s translational potential in the context of human reproduction.

**Supplementary Information:**

The online version contains supplementary material available at 10.1007/s10815-025-03720-1.

## Introduction

Ageing is a battle fought at the cellular level, and the human oocyte, a powerhouse of reproductive capability, is one of the first cells to experience age-associated functional decline [1]. At the heart of cellular vitality lies nicotinamide adenine dinucleotide (NAD⁺), a coenzyme that regulates a wide range of essential biological processes, including mitochondrial function, oxidative stress response, and cellular signalling [2].

NAD⁺, known as the key molecule in redox reactions, acts as an electron carrier in metabolic pathways such as glycolysis, the tricarboxylic acid (TCA) cycle, and oxidative phosphorylation, where it helps in the production of ATP [2, 3]. Apart from its role in energy metabolism, NAD⁺ is also known to be the major substrate for several families of enzymes, including sirtuins, poly(ADP-ribose) polymerases, and cyclic ADP-ribose synthetases, all of which contribute to genomic stability, repair of DNA, and adaptation to stress conditions [3]. However, the levels of NAD⁺ decline with age and metabolic stress, causing widespread cellular dysfunction. This has been observed in many of the species, from worms to rodents and humans, and in several of the tissues, from skeletal muscle to liver, brain, and skin [4–6].

Oocytes have special sensitivity towards mitochondrial dysfunction as they depend on oxidative phosphorylation for ATP production. Glycolytic compensation for mitochondrial dysfunction, noted in other cell types, cannot help oocytes since they need mitochondria-mediated energy for meiotic maturation as well as spindle formation, chromosomal segregation, and early embryonic development [7].

### Nicotinamide mononucleotide

Given the central role of NAD⁺ in cellular metabolism and stress resistance, there has been growing interest in its potential application as an adjuvant intervention in reproductive biology. NAD⁺ homeostasis is regulated through biosynthetic and salvage pathways, where precursors such as nicotinamide riboside (NR) and nicotinamide mononucleotide (NMN) serve as intermediates for NAD⁺ synthesis [8]. Among these, NMN has gained particular attention due to its ability to rapidly restore intracellular NAD⁺ levels, enhancing mitochondrial function, DNA repair, and cellular resilience in various ageing and metabolic models.

Recent studies suggest that NMN supplementation may improve age-related mitochondrial decline in murine models by activating sirtuin-dependent pathways that regulate mitochondrial biogenesis, oxidative stress defence, and apoptosis [9]. Sirtuins, particularly sirtuin 1 (SIRT1) and sirtuin 3 (SIRT3), are NAD⁺-dependent deacetylases that modulate key metabolic and stress-response pathways. SIRT1 is involved in maintaining genomic stability by modulating chromatin structure, suppressing DNA damage accumulation, and regulating the activity of key DNA repair proteins. SIRT3 is a mitochondrial deacetylase that plays a crucial role in maintaining oxidative balance [10, 11]. It regulates the deacetylation of key enzymes involved in the electron transport chain, TCA cycle, and antioxidant mechanisms. Given that NAD⁺ depletion impairs sirtuin activity, it has been hypothesised that NMN supplementation could restore sirtuin-mediated pathways, thereby improving mitochondrial health and enhancing oocyte quality [9, 10].

This systematic review aims to explore recent findings on the potential application of NMN in female reproduction, particularly in enhancing oocyte quality prior to retrieval. Furthermore, we aim to explore the transcriptomic fingerprint of human oocytes at different maturity stages to characterise the expression patterns associated with NAD⁺ metabolism and establish future research directions in this field.

## Methods

The search protocol was registered at PROSPERO (ID number CRD420251017045). For the transcriptomic analysis of human oocytes, ethical approval was granted from the London – Surrey Research Ethics Committee (reference:18/LO/1849; Go project ID 253968).

### Design and search strategy

A systematic review was conducted to identify, screen, and synthesise relevant studies in a structured and replicable manner. The search was performed across three major databases: Medline, Embase, and Scopus to identify studies investigating NAD⁺ metabolism, NMN supplementation, and their impact on oocyte quality. For Scopus, subject areas were limited to the following: Biochemistry, Genetics and Molecular Biology, Medicine, Chemistry, Immunology and Microbiology, and Health Professions.

The search strategy employed free-text keywords, aligning with standardised terminology where applicable, to ensure broad retrieval of relevant publications. Boolean operators (AND/OR) and parentheses were used to combine the keywords and refine the search results. The search terms used were as follows: (NAD metabolism OR NAD⁺ metabolism OR NAD OR nicotinamide adenine dinucleotide OR NMN supplementation OR nicotinamide mononucleotide) AND (oocyte OR fertility OR ovarian reserve OR female reproductive health) AND (gene expression OR transcriptomic OR mRNA OR RNA sequencing OR differential expression OR gene regulation OR pathway).

The study selection process followed the Preferred Reporting Items for Systematic Reviews and Meta-Analysis (PRISMA) guidelines [12]. All retrieved studies were independently screened by two reviewers (H.N and X.V.G). Publications between 1 st January 2015 and 31 st October 2024 were reviewed. No restrictions were applied regarding time or species, but only English-language transcripts were reviewed. Only original sources, such as peer-reviewed journal articles, were included to ensure the reliability of the results. The most recent search was conducted on 11th October 2024.

### Quality assessment

The quality and risk of bias of the included studies was evaluated using the National Heart, Lung, and Blood Institute (NHLBI)-National Institutes of Health (NIH) quality assessment tool for case–control studies by two reviewers (H.Y. and X.V.G.). This tool comprises 12 criteria assessing study design, methodology, and reporting quality. Each criterion was scored as follows: 1 (yes), 0.5 (partially), or 0 (no or not applicable), yielding a total score ranging from 0 to 12. Studies scoring 0–4 were classified as poor, those scoring 4.5–8 as fair, and those scoring 8.5–12 as good. Only studies rated as “good” by both independent reviewers were included in the final synthesis, resulting in seven studies selected for data extraction.

### Data extraction and synthesis

Table 1 provides a summary of the study characteristics, including the study country, study type, experimental groups, NAD⁺-related genes investigated, supplementation or intervention used, and gene expression changes. Data from the included studies were analysed using a thematic analysis approach, allowing the integration of findings across different study designs. Following data extraction, pathway annotation analysis of the NAD⁺-related genes was conducted using the Reactome database [13]. Overrepresentation analysis was performed against Reactome version 91.

**Table 1:** Data extracted from selected studies. Aim, methodology, and findings are described for each of the studies, together with their corresponding NHLBI-NIH score

Author(s), date, country	Study aim	Species/sample type	Experimental groups	Supplement/intervention used	Gene(s) of interest	NHLBI-NIH tool score
Bertoldo et al., (2018) [17], Australia	To investigate whether maternal and post-weaning HFD-induced changes in oocyte-secreted factor signalling and ovarian function in female offspring could be ameliorated by exercise and NMN supplementation	Female C57BL/6 mice (*Mus musculus*)	1. Chow maternal diet, chow offspring diet, no intervention (control) 2. Chow maternal diet, HFD offspring diet, no intervention 3. HFD maternal diet, chow offspring diet, no intervention 4. HFD maternal diet, HFD offspring diet, no intervention 5. HFD maternal diet, chow offspring diet, exercise intervention 6. HFD maternal diet, chow offspring diet, NMN intervention 7. HFD maternal diet, HFD offspring diet, exercise intervention 8. HFD maternal diet, HFD offspring diet, NMN intervention	1. Exercise intervention Type: treadmill running Duration: 9 weeks (starting at 9 weeks of age) Frequency: 6 days per week 2. NMN supplementation Form: intraperitoneal (i.p.) injections of NMN Dose: 500 mg/kg body weight per day Duration: 2.5 weeks (from 16 to 18.5 weeks of age)	**In COCs:** *Gdf9*, *Bmp15*, *Amhr*, *Mpc1*, *Sirt1* **In the ovary:** *Gdf9, Bmp15, Fshr, Sirt3, Nfkb1, Insr, Sirt1, Prkaa2, Cry1*	9
Guo et al., (2024) [18], China	To investigate the effects of NMN on oocyte maturation in STZ-induced diabetic mice and explore the underlying mechanisms by assessing mitochondrial function, oxidative stress, DNA damage, actin dynamics, meiotic defects, and histone modifications	Female ICR mice (*Mus musculus*)	1. Control group—non-diabetic mice injected with sodium citrate buffer; oocytes matured without NMN 2. Diabetic group (T1D mice without NMN)—diabetic mice injected with STZ; oocytes matured without NMN 3. Diabetic + NMN group (T1D Mice with NMN)—diabetic mice injected with STZ; oocytes matured with NMN supplementation	1. NMN supplementation Form: NMN in solution, diluted in M16 medium Dose: 1 μM final concentration in the culture medium Duration: 14–16 h (during in vitro maturation of oocytes)	**In oocytes:** *Sirt1, Sirt3, Drp1, Opa1, Mfn2, Sod1*	10
Huang et al., (2022) [28], China	To investigate whether long-term NMN supplementation can counteract age-related ovarian decline in mice by enhancing mitophagy in granulosa cells, improving ovarian follicle reserve, endocrine function, and mitochondrial biogenesis, while reducing cellular senescence	Female ICR mice (*Mus musculus*)	1. 4 W group: young control (saline) 2. 8 W group: early reproductive age control (saline) 3. 12 W group: peak reproductive age control (saline) 4. 24 W group: mid-reproductive age control (saline) 5. 40 W group: pre-ageing control (saline) 6. 60WC group: aged control (saline from 40 to 60 weeks) 7. 60WN group: NMN-treated group (NMN from 40 to 60 weeks)	1. NMN supplementation Form: oral (drinking water) Dose: 0.5 mg/mL in drinking water Duration: 20 weeks (from 40 to 60 weeks of age)		9
Jiang et al., (2023) [25], China	To investigate the reproductive toxicity of BBP in mouse oocytes and assess whether NMN supplementation can counteract BBP-induced oxidative stress, mitochondrial dysfunction, and apoptosis	Female ICR mice (*Mus musculus*)	1. Control group (no BBP, no NMN) 2. Low-dose BBP group (0.5 mg/kg BW/day BBP) 3. High-dose BBP group (1.5 mg/kg BW/day BBP) 4. BBP + NMN group (1.5 mg/kg BW/day BBP + 200 mg/kg BW/day NMN) 5. In vitro oocyte culture groups (control vs BBP-treated oocytes) 6. In vivo fertilisation groups (control vs BBP-exposed vs BBP + NMN)	1. NMN supplementation Form: intraperitoneal (i.p.) injection Dose: 200 mg/kg body weight per day Duration: 8 consecutive days, administered alongside BBP exposure	**In oocytes:** *Crisp1, Scd3, Kif18b, Atp6v1c2* **In the ovary:** *P16, Pgc-1α, Nrf-1, Lc3b, Lamp-1, Clpp, Ctsd*	9
Li et al., (2023) [29], China	To investigate whether NMN supplementation could mitigate the effects of ageing in porcine oocytes and improve subsequent embryonic development	Porcine oocyte	1. Fresh oocytes (0 h)—control group 2. Aged oocytes (24 h, 48 h)—untreated ageing groups 3. NMN-treated aged oocytes (1, 10, 100 μmol/L)—tested for rescue effects 4. Embryo development groups—fresh, aged (24 h, 48 h), and NMN-treated aged oocytes assessed for blastocyst formation	1. NMN supplementation Form: dissolved in porcine zygote medium (pZM-3) Dose: 1, 10, and 100 μmol/L (100 μmol/L was optimal) Duration: applied to oocytes during in vitro ageing for 24 or 48 h	**In oocytes:** *Sod1, Cat, Bax, Bcl-2* **In blastocysts:** *Nanog, Oct4, Sox2*	9.5
Wang et al., (2022) [19], China	To investigate whether NMN supplementation could improve oocyte quality and reproductive outcomes in obese female mice by restoring mitochondrial function, reducing oxidative stress, and alleviating ovarian inflammation	Female C57BL/6 J mice (*Mus musculus*)	1. ND (normal diet) group—mice fed a normal diet, received PBS injections (healthy control) 2. HFD group—mice fed a high-fat diet for 12 weeks, received PBS injections (obese control) 3. HFD + NMN group—mice fed a high-fat diet for 12 weeks, received NMN injections (obese treatment group)	1. NMN supplementation Form: Intraperitoneal (i.p.) injection Dose: 200 mg/kg body weight per day Duration: 10 consecutive days	**In the ovary:** *Lhx8, Bmp4, Adgre1, Ccl2, TNF-α, Gal-3, Clec10a, IL-10* **In oocytes:** *Bax, Sod1*	9
Xu et al., (2023) [26], China	To investigate whether NMN, BER, and COR improve the survival and developmental potential of vitrified bovine oocytes by reducing lipid accumulation, oxidative stress, ER stress, and apoptosis while enhancing mitochondrial function	Bovine oocyte	1. Fresh control group—untreated, non-vitrified oocytes 2. Vitrification control group—vitrified oocytes with no supplementation 3. NMN groups—oocytes treated with NMN during IVM 4. BER groups—oocytes treated with BER during IVM 5. COR groups—oocytes treated with COR during IVM 6. Optimised treatment groups—the most effective concentrations (1 µM NMN, 2.5 µM BER, and 1 µM COR) used for final comparisons	1. NMN, BER, COR Supplementation Form: dissolved in IVM medium (M199) Dose: 0.1, 1, 10, 100 µM for MNM; 1.25, 2.5, 5, 10 µM for BER; 0.1, 1, 10, 100 µM for COR Duration: 22–24 h during IVM	**In oocytes:** *Srebp1, Fabp3, Pparg, Xbp1, Atf4, Grp78, Mfn1, Mfn2, Fis1, Drp1, Cat, Gpx1, Sod1, Bax, Bcl2*	8.5

### Transcriptomic analysis

For single-oocyte RNA sequencing, a total of 50 oocytes were obtained from 28 female participants (age 27–39). The sample collection followed specific exclusion criteria, which included retrieval of fewer than four metaphase II oocytes (indicating a suboptimal response), body mass index (BMI) exceeding 30 kg/m^2^, presence of uterine abnormalities, endometriosis, polycystic ovary syndrome (PCOS), and history of recurrent pregnancy loss. Out of the collected samples, 46 were successfully sequenced from 25 patients (Supplementary Table 1). These included 14 germinal vesicle (GV) stage oocytes, 9 metaphase I (MI) oocytes, and 23 metaphase II (MII) oocytes.

Library preparation was conducted using the NEBNext Single Cell/Low Input RNA Library Prep Kit for Illumina (New England BioLabs®, USA, #E6420), following the manufacturer’s protocol with a single modification: NEBNext Adaptors were replaced with IDT xGen UDI-UMI adaptors. Sequencing was performed on the NextSeq 500 platform using a v2.5 High Output 75-cycle kit, generating 20,024,906 reads. Demultiplexing and FASTQ file generation were carried out using Illumina’s bclConvert software (v3.7.5). Quality control and downstream bioinformatics analyses were performed on the Galaxy platform [14]. Differential gene expression analysis was conducted using the Limma-voom tool [15], based on the sample metadata provided. The detectable fold change for comparisons was determined following the method described by Hart et al., using a statistical power of 0.9 and a false discovery rate (FDR) threshold of 0.01 [16]. The detectable fold change for GV-MI, GV-MII, and MI-MII comparisons was 5.61, 3.89, and 4.32, respectively.

## Results

### Search strategy

**Fig. 1:**
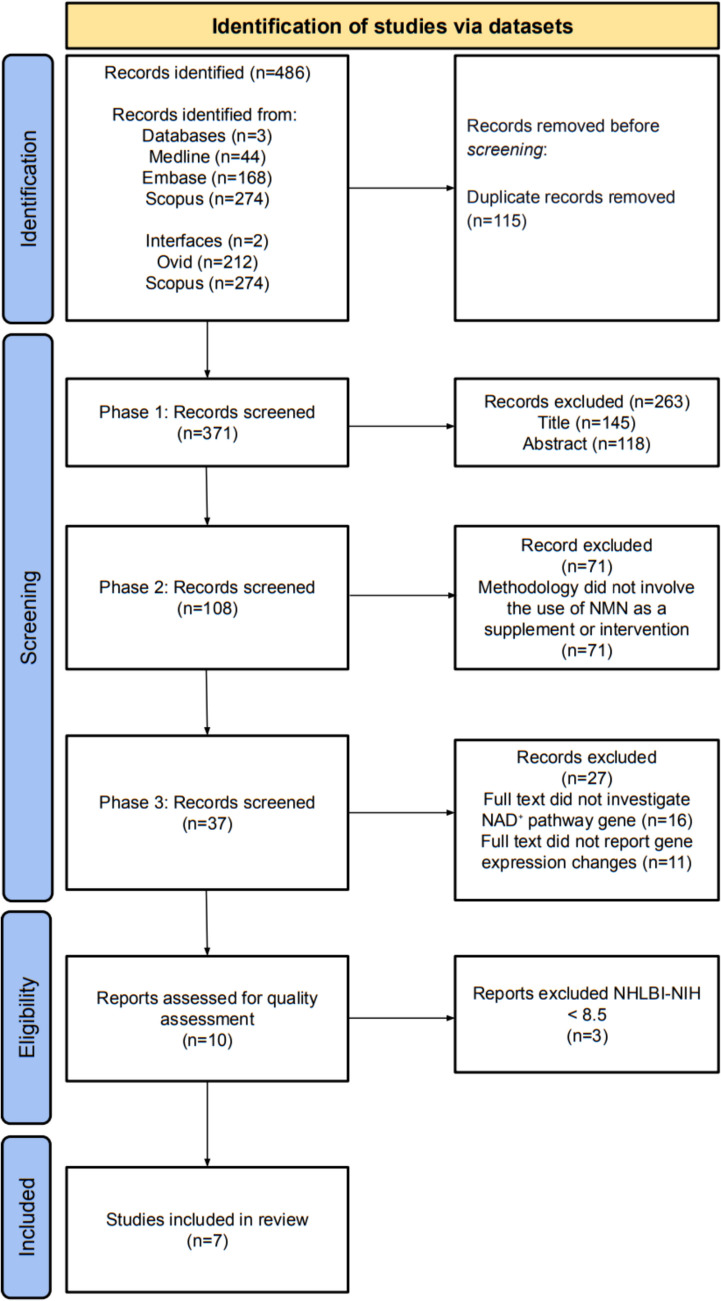
PRISMA information flow diagram. Summarised the detailed database searches, the number of studies screened, and the full texts retrieved

A total of 486 records were identified. After duplicate removal, 371 unique records remained. Following phase one screening, 108 studies were selected for further evaluation. Phase two screening narrowed the selection to 37 publications. Phase three screening resulted in 10 studies undergoing quality assessment using the NHLBI-NIH assessment tool. Of these, seven original research articles met the inclusion criteria and were selected for data extraction and synthesis (Fig. 1).

### Study characteristics

Of the seven studies, six were conducted in China and one in Australia. The NHLBI-NIH quality assessment scores for the quantitative studies ranged from 8.5 to 10. All studies employed controlled experimental animal models: three used an in vivo approach, two adopted an in vitro approach, and two combined in vivo and in vitro methodologies. Five studies investigated female mice (*Mus musculus*) and their oocytes, with two using female C57BL/6 mice and three using female ICR mice. The remaining studies focused on porcine and bovine oocytes.

The studies utilised distinct age models to represent various stages of reproductive development and ageing. Four studies focused on early-life models, using female mice aged 3–6 weeks to investigate the effects of NMN intervention following early exposures such as type 1 diabetes (T1D), environmental toxins, or high-fat diets (HFD). One study administered NMN to 16-week-old mice, representing young adulthood. Later-stage interventions were employed in two studies: one examined reproductive ageing across a 4- to 60-week continuum, introducing NMN supplementation at 40 weeks, while the other modelled post-ovulatory ageing by culturing porcine oocytes for 24 and 48 h. Collectively, these models enabled the examination of molecular and functional changes across a broad reproductive timeline.

### Systematic analysis theme overview

The studies used various physiological and environmental models, categorised into three themes: intrinsic stress models, exogenous stress models, and the effect of ageing on oocyte quality. Intrinsic metabolic dysregulation was examined through HFD exposure and T1D models to understand its impact on oocyte and ovarian function. Exogenous stress was studied via environmental toxins and cryopreservation protocols, highlighting external factors affecting oocyte quality. Ageing was investigated using both in vivo and in vitro models to explore molecular and cellular changes in reproductive ageing. All seven studies examined gene expression changes, analysing 49 unique genes across cumulus–oocyte complexes (COCs), ovaries, oocytes, and blastocysts.

### Transcriptomic analysis in light of previous work

We identified nine hundred differentially expressed genes when comparing GV to MII-stage oocytes (756 upregulated and 144 downregulated). Gene ontology (GO) analysis of the differentially expressed genes revealed that GV oocytes (compared to MII) had enriched terms related to organelle organisation, cellular component biogenesis, and mitochondrial function (GO, biological processes) (Supplemental Fig. 1). With regard to cellular component GOs, there were enriched terms for intracellular organelles and structure and intracellular membrane-bounded organelles. Looking at molecular function, the most overrepresented categories were molecular function and binding.

**Fig. 2:**
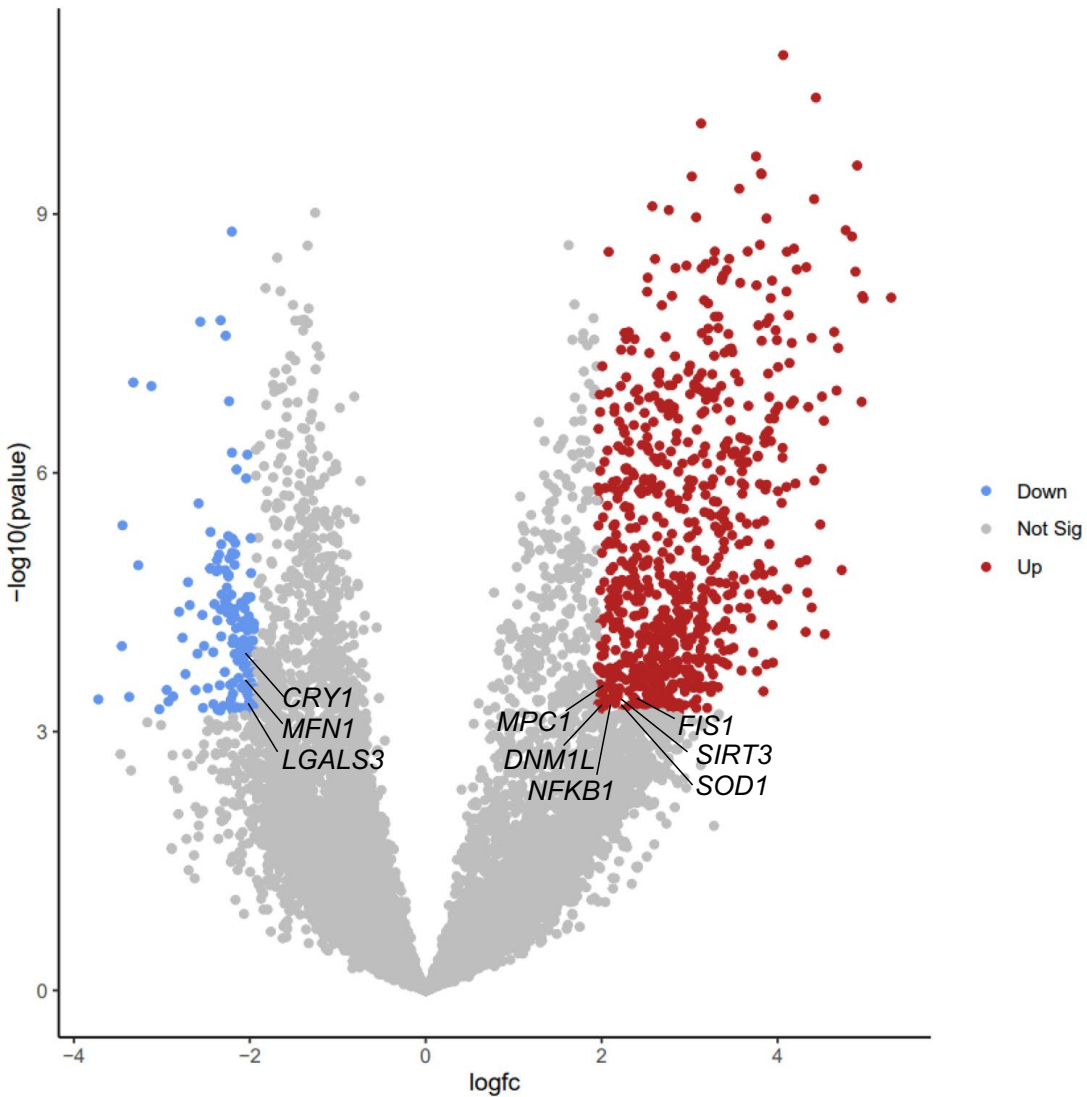
Differential gene expression between GV and MII oocytes. Volcano plot displaying transcriptomic differences between GV and MII oocytes. The *x*-axis represents the log_2_ fold change (logFC), and the *y*-axis shows the − log_10_(*p*-value). Genes significantly downregulated in GV oocytes are shown in blue, upregulated genes in red, and non-significant genes in grey. The nine annotated genes were identified through a systematic review and exhibit statistically significant differential expression: six are upregulated (*FIS1*, *SIRT3*, *SOD1*, *MPC1*, *DNM1L*, *NFKB1*) and three are downregulated in GV (*CRY1*, *MFN1*, *LGALS3*)

**Fig. 3:**
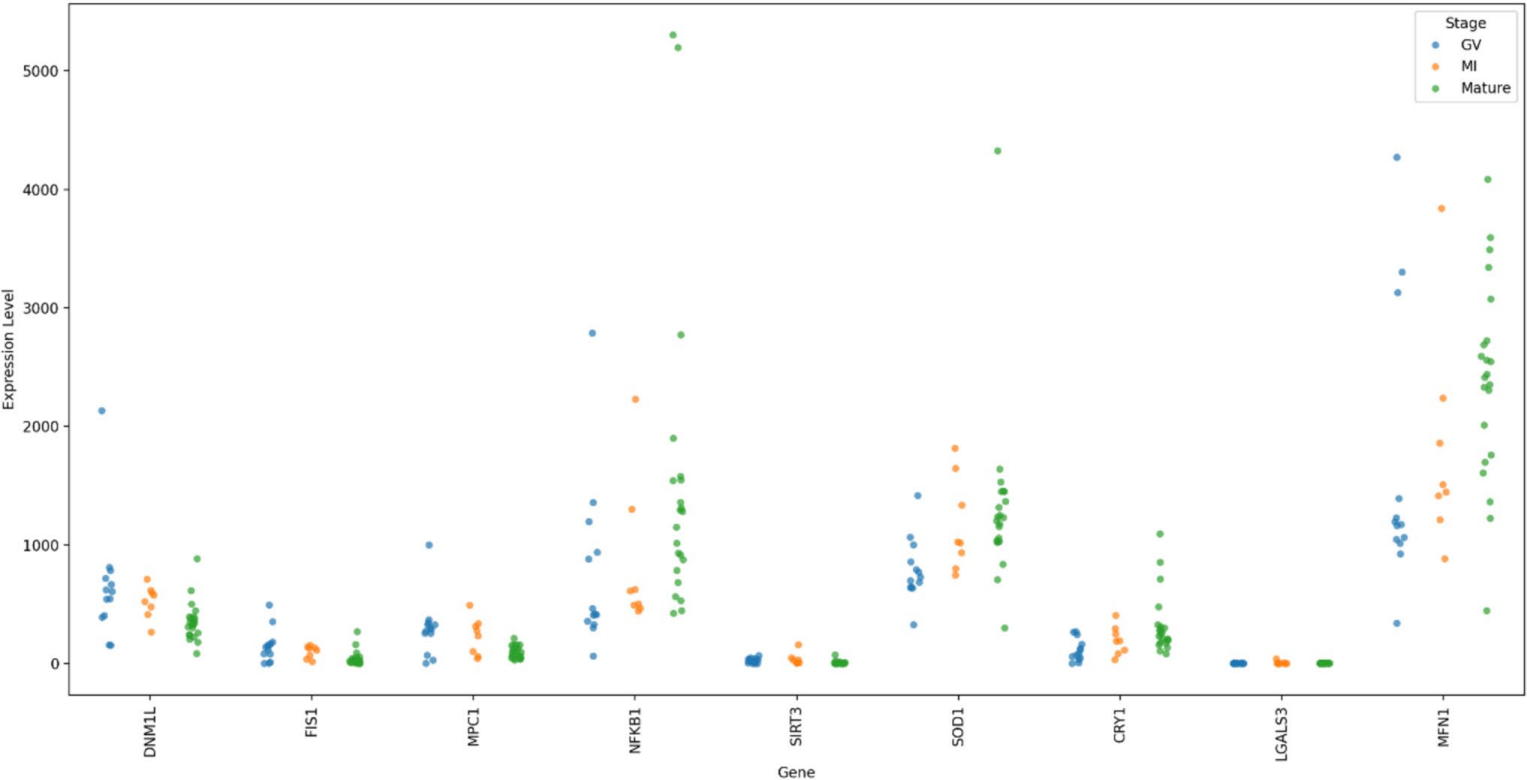
Distribution of gene expression levels across different genes and developmental stages. Strip chart comparison of gene counts per sample between stages, highlighting stage-specific expression patterns

Forty-nine genes were identified through the systematic review. Single-cell analysis determined that nine exhibited significant differential expression in the comparison between the GV and MII stages (FDR *P* < 0.05) (Fig. 2). Among these, *DNM1L*, *FIS1*, *MPC1*, *NFKB1*, *SIRT3*, and *SOD1* were upregulated in the GV stage relative to MII, while *CRY1*, *LGALS3*, and *MFN1* were downregulated in GV compared to MII. Only one gene, *SIRT3*, demonstrated a significant difference in the MI versus MII comparison, showing upregulation in MI relative to MII (FDR *p*-value < 0.05) (Fig. 3).

## Discussion

### Intrinsic disruptors and NMN-mediated response

Different studies looked at metabolic disruption models, namely maternal and offspring following a high-fat diet (HFD) exposure, maternal HFD-induced obesity, and maternal type 1 diabetes (T1D) [17–19]. One feature shared among all of them was mitochondrial dysregulation. In T1D-exposed oocytes, mitochondrial regulators *Drp1*, *Opa1*, and *Mfn2* were downregulated, alongside NAD⁺-dependent deacetylases *Sirt1* and *Sirt3*, indicating impaired energy metabolism.

Oxidative stress and immune dysregulation were consistently observed across models. In both T1D and HFD-exposed oocytes, *Sod1*, a crucial antioxidant enzyme, was downregulated, indicating increased oxidative stress. In the HFD-induced obesity model, *Bax* was upregulated, suggesting heightened apoptotic vulnerability. Inflammatory responses varied by tissue and model. Maternal HFD exposure elevated ovarian expression of pro-inflammatory genes (*Adgre1*, *Ccl2*, *Tnf*, *Lgals3*) and suppressed anti-inflammatory genes (*Clec10a*, *Il10*), indicating a shift towards a pro-inflammatory state. *Nfkb1* was downregulated with maternal HFD, while *Lnsr* was upregulated in offspring exposed to HFD, potentially reflecting adaptation to chronic metabolic stress.

NMN supplementation showed partial reversal of transcriptional dysregulation. In COCs from HFD-exposed mice, NMN normalised *Gdf9* and *Mpc1* expression, though *Bmp15* remained unchanged. In ovarian tissue, NMN upregulated *Gdf9*, *Prkaa2*, *Cry1*, and *Sirt1*, suggesting improved follicular and metabolic function. However, *Bmp15*, *Fshr*, *Sirt3*, and *Nfkb1* were unaffected, indicating selective gene responsiveness. Changes in *Gdf9* and *Bmp15* expression following maternal and offspring high-fat diet exposure suggest disrupted follicular signalling and accumulating metabolic stress across generations, underscoring the lasting impact of early-life nutrition on reproductive health [20–23].

In the T1D model, NMN restored mitochondrial regulatory genes (*Sirt1*, *Sirt3*, *Drp1*, *Opa1*, *Mfn2*) and increased *Sod1*, enhancing oxidative stress resilience and energy balance. These findings align with previous work identifying Sirt1-mediated pathways as key regulators of reproductive ageing and metabolic homeostasis [24]. In the HFD model, NMN reinstated *Lhx8* and *Bmp4* expression and modulated inflammatory signalling by downregulating pro-inflammatory and upregulating anti-inflammatory mediators. Additionally, *Bax* was reduced and *Sod1* increased in oocytes, indicating improved mechanisms against apoptosis and oxidative damage.

### Extrinsic disruptors and NMN-mediated response

**Fig. 4:**
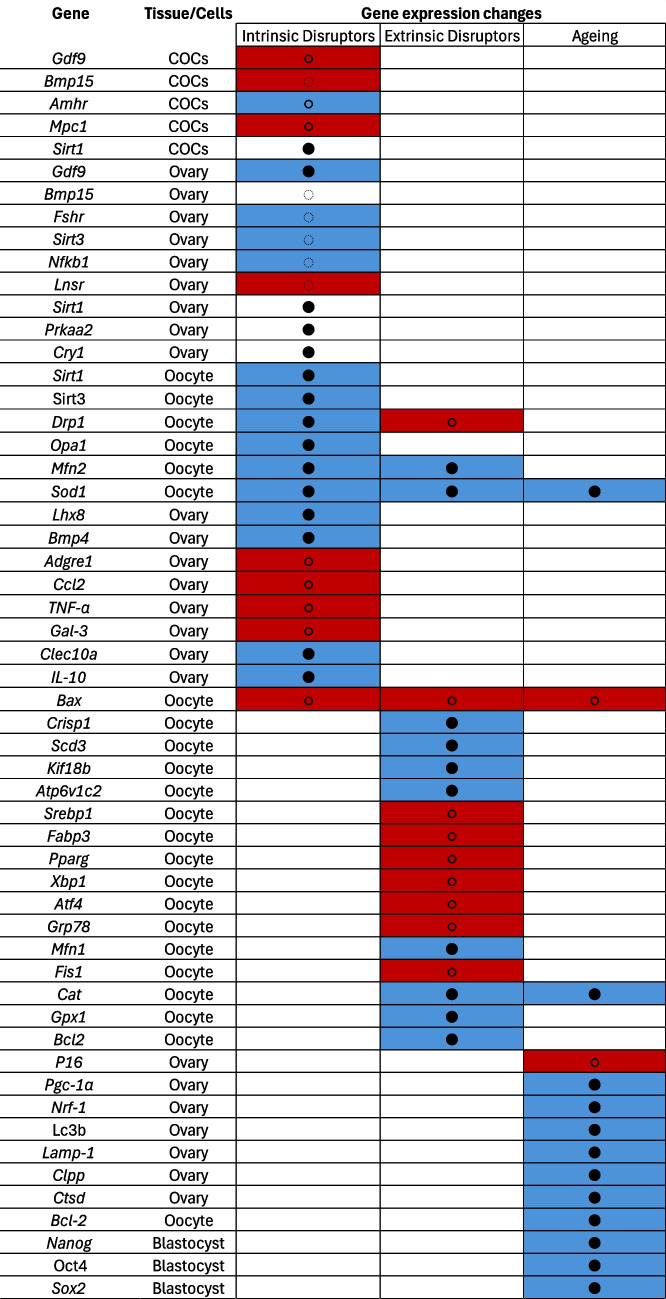
Gene expression changes and NMN-mediated rescue. Each cell represents the average effect on gene expression, downregulation (blue), upregulation (red), and no change (white). Overlaid dots indicate the effect of NMN treatment: empty dots represent NMN-induced downregulation, black dots represent NMN-induced upregulation, and dotted dots represent no change in gene expression. This visualisation highlights genes whose dysregulation may be reversed or modulated by NMN treatment

Oocytes are particularly susceptible to exogenous stressors, including environmental toxicants and procedural interventions such as cryopreservation. Two studies that modelled these stressors through exposure to butyl benzyl phthalate (BBP) and cryopreservation-induced damage highlight convergent molecular mechanisms of oocyte disruption [25, 26]. These include dysregulated lipid metabolism, mitochondrial dysfunction, oxidative stress, and apoptosis. In both models, supplementation with NMN emerged as a promising intervention to counteract these effects and restore oocyte function (Fig. 4).

Across both stress models, transcriptomic analyses revealed significant alterations in gene expression involved in mitochondrial bioenergetics and structural integrity. In BBP-exposed oocytes, there was notable downregulation of genes critical for mitochondrial and cytoskeletal regulation, including *Crisp1*, *Scd3*, *Kif18b*, and *Atp6v1c2*. This aligns with previous findings suggesting that environmental endocrine disruptors impair mitochondrial function and oxidative balance, leading to poor oocyte quality and reduced fertilisation potential [27]. Similarly, cryopreservation induced mitochondrial fragmentation, as evidenced by decreased expression of fusion-related genes (*Mfn1*, *Mfn2*) and upregulation of fission-associated genes (*Fis1*, *Drp1*), indicating a breakdown in mitochondrial homeostasis that compromises energy production and cellular viability. NMN supplementation restored the expression of mitochondrial genes in both models, suggesting a conserved mechanism through which NMN supports mitochondrial dynamics and function (Fig. 4).

Lipid metabolism also emerged as a commonly affected pathway. Cryopreserved oocytes showed increased expression of *Srebp1*, *Fabp3*, and *Pparg*, indicative of enhanced lipid synthesis and storage. Although BBP-induced alterations in lipid metabolism were less extensively characterised, the downregulation of *Scd3*, a key enzyme in lipid desaturation, similarly points to metabolic dysregulation. In both contexts, NMN treatment normalised the expression of lipid-associated genes.

Oxidative stress and apoptosis were consistently elevated across both models. BBP exposure and cryopreservation each led to increased oxidative damage and compromised antioxidant defences. In cryopreserved oocytes, this was marked by reduced expression of *Cat*, *Gpx1*, and *Sod1*, alongside elevated pro-apoptotic *Bax* and decreased anti-apoptotic *Bcl2*. NMN supplementation reversed these molecular signatures, enhancing antioxidant gene expression and modulating apoptotic pathways. These protective effects were mirrored in the BBP model, where NMN restored expression of genes involved in redox balance (*Scd3*) and cytoskeletal organisation (*Crisp1*, *Kif18b*).

### The effect of ageing and NMN-mediated response

Age-related stress in ovarian and oocyte systems leads to cellular senescence, mitochondrial dysfunction, oxidative stress, impaired autophagy, and reduced developmental potential. Across ovarian, oocyte, and blastocyst models, a consistent pattern emerges in which ageing disrupts cellular homeostasis, while NMN supplementation reverses these effects by enhancing mitochondrial metabolism, antioxidant defences, and proteostasis [28, 29].

In aged ovarian tissue, elevated expression of *P16*, a hallmark of cellular senescence, was particularly evident in granulosa cells and the corpus luteum. Concurrently, key regulators of mitochondrial biogenesis, *Pgc-1α* and *Nrf-1*, were downregulated, indicating compromised mitochondrial function and reduced metabolic capacity, consistent with previous studies [30]. Aged oocytes exhibited significant downregulation of antioxidant genes *Sod1* and *Cat*, contributing to increased oxidative stress. Apoptotic signalling was also perturbed, with elevated *Bax* and reduced *Bcl2* expression, resulting in a lower *Bcl2/Bax* ratio and heightened apoptotic susceptibility. These molecular signatures reflect a broader decline in cellular resilience across reproductive tissues.

NMN supplementation effectively reversed many of these age-associated alterations (Fig. 4). In ovarian tissue, NMN reduced *P16* expression and restored *Pgc-1α* and *Nrf-1* levels, indicating reduced senescence and improved mitochondrial biogenesis. It also enhanced autophagic and lysosomal function, as evidenced by the recovery of *Lc3b*, *Lamp1*, *Clpp*, and *Ctsd* expression, supporting improved proteostasis, consistent with previous findings [31]. In oocytes, NMN restored antioxidant gene expression and rebalanced apoptotic signalling by increasing *Bcl2* and decreasing *Bax*.

These molecular improvements translated into enhanced developmental potential. Blastocysts derived from aged oocytes exhibited downregulation of pluripotency-associated genes *Nanog*, *Oct4*, and *Sox2*, reflecting impaired transcriptional activity essential for embryogenesis. NMN supplementation restored the expression of these genes, suggesting improved developmental competence and early embryonic viability.

### Transcriptomic insights

The enriched pathways identified in the transcriptomic analysis of human oocytes, particularly those related to mitochondrial function, oxidative stress, and cellular organisation,align with key biological processes modulated by NMN in preclinical models. This convergence supports the translational relevance of NMN supplementation and suggests that the molecular mechanisms observed in animal studies may be conserved in human oocyte maturation.

The upregulation of mitochondrial genes, particularly *DNM1L, FIS1, MPC1*, and *SIRT3*, indicates that mitochondrial remodeling is highly active in immature oocytes. Dynamin-1-like protein (DNM1L) and mitochondrial fission 1 protein (FIS1) are key regulators of mitochondrial fission, a process essential for redistributing mitochondrial content and ensuring that only functional mitochondria are inherited by the developing embryo [32, 33]. Their upregulation in GV-stage oocytes suggests that mitochondrial fragmentation plays a crucial role at this stage, enhancing bioenergetic efficiency and facilitating the removal of dysfunctional mitochondria. Moreover, the upregulation of the mitochondrial pyruvate carrier 1 gene (MPC1) suggests that pyruvate metabolism is highly active during the GV stage, supporting ATP production via oxidative phosphorylation. Given the established link between oocyte competence and ATP availability, this finding aligns with previous research demonstrating that metabolic regulation is fundamental to oocyte maturation [34]. The increased expression of *SIRT3* further reinforces this concept. *SIRT3* is critical for mitochondrial metabolism, ATP production, and oxidative stress defence [10]. The sustained expression of *SIRT3* in MI oocytes, compared with MII, suggests that its regulatory functions persist throughout oocyte maturation.

The differential expression of oxidative stress–related genes provides further insight into the metabolic shifts occurring during oocyte development. The higher expression of *SOD1* in GV-stage oocytes compared with MII suggests that early-stage oocytes may require enhanced oxidative stress defence. Superoxide dismutase 1 (SOD1) is a key enzyme that neutralises ROS, preventing oxidative damage that could compromise oocyte viability [35]. The increased expression of *NFKB1* in GV-stage oocytes further supports this, as nuclear factor kappa B (*NF-κB*) is a major regulator of cell survival, inflammatory responses, and stress adaptation [36]. The activation of NF-κB signalling in early-stage oocytes may be critical for maintaining cellular integrity as they progress through maturation.

Single-cell analysis of larger and more biologically diverse cohorts could extend these findings and further investigate age-related differences, helping to determine whether NMN-rescue pathways observed in animal models are conserved in humans.

## Conclusion

The systematic review examined the potential of NMN supplementation to enhance oocyte quality under diverse biological stressors. A synthesis of seven preclinical studies demonstrated that NMN consistently improved oocyte and ovarian function across models of metabolic, exogenous, and age-associated stress.

### Methodological variability and translational challenges

There was considerable variability in NMN administration methods, posing a challenge for cross-study comparisons. Intraperitoneal injection allows rapid systemic absorption but differs from physiological NMN intake in humans. Oral supplementation more closely replicates dietary NMN consumption but is influenced by metabolism and gut microbiota [37, 38]. In vitro supplementation ensures controlled exposure but lacks systemic regulatory factors, including NMN transport and metabolism [39]. These differences underscore the need for standardised administration protocols to enhance comparability across studies.

While most NMN studies rely on murine models, species-specific differences in oocyte maturation and NAD⁺ metabolism may limit their direct relevance to human fertility. Notably, variations in NAD⁺ biosynthesis pathways could modulate NMN’s efficacy across species. Mice exhibit higher metabolic rates and shorter reproductive lifespans, which may amplify NMN’s perceived benefits in age-related fertility decline [39, 40]. In contrast, bovine and porcine oocytes have longer maturation windows, more closely mirroring human reproductive timelines, yet their metabolic responses to NMN remain poorly characterised [41]. Future research should determine whether NMN’s effects are conserved across species and whether metabolic adaptations influence its efficacy in human oocytes.

A substantial disparity exists between NMN dosages used in in vivo and in vitro studies. In vivo experiments typically require doses of 200–500 mg/kg/day to mitigate systemic stressors such as obesity and environmental toxins, whereas in vitro models employ significantly lower concentrations (1–100 µM) to support oocyte maturation without inducing cytotoxicity. While high in vivo doses may be necessary to achieve sufficient systemic NAD⁺ enhancement, they could also activate metabolic compensation mechanisms or oxidative stress responses, potentially confounding results [42]. According to Reagan-Shaw et al., 2008, 300 mg/kg/day in mice is equivalent to approximately 24 mg/kg/day in humans, based on body surface area normalisation [43]. In humans, preliminary clinical trials have primarily focused on assessing NMN’s safety [44–46]. For instance, a study involving healthy Japanese men demonstrated that single-dose administration of up to 500 mg of NMN was well tolerated and effectively increased NAD⁺ levels [45]. However, the long-term safety of such doses remains uncertain across populations, highlighting the need for further investigation into dose–response relationships and potential unintended effects.

A limitation of this review is the geographic concentration of the included studies. Research environments, animal handling protocols, and dietary or environmental exposures can vary significantly across regions, potentially influencing experimental outcomes. These factors can introduce bias and limit the generalizability of the findings. Future studies from diverse geographic and institutional settings are essential to validate the reproducibility and broader applicability of NMN’s effects on oocyte quality. *On Fertility: Can NMN save the egg?*

Metabolic dysregulation and ageing intersect through shared molecular pathways that impair oocyte quality and ovarian function, primarily via disruptions in folliculogenesis, mitochondrial metabolism, oxidative stress, inflammation, and apoptosis. Our review highlights that NMN supplementation in animal models consistently modulates key genes such as *Gdf9*, *Sirt1*, and *Sod1*, though its effects are not universally observed; highlighting both its targeted potential and the complexity of reproductive decline. Notably, NMN’s ability to restore mitochondrial integrity, redox balance, and cellular resilience positions it as a promising multifaceted intervention. Improvements in oocyte competence and developmental potential across both metabolic and ageing models suggest broad therapeutic relevance.

Complementing these findings, transcriptomic analysis of human oocytes revealed maturation-dependent regulatory shifts, indicating a potential window for intervention. These insights support further investigation into NMN’s clinical utility, with emphasis on standardised, large-scale studies to determine optimal dosing, safety, and efficacy in human fertility contexts.

## Supplementary Information

Below is the link to the electronic supplementary material.

ESM 1 (DOCX 20.9 KB)

ESM 2 (DOCX 753 KB)

## Data Availability

The datasets generated during this study are not publicly available due to participant confidentiality; however, they may be obtained from the corresponding author upon submission of a reasonable request.
