# Supplementary material for: NMN supplementation as a strategy to improve oocyte quality: a systematic review and transcriptomic analysis
Source: J Assist Reprod Genet. 2025 Oct 29;43(1):51–65. doi: 10.1007/s10815-025-03720-1 (PMC12831783; doi:10.1007/s10815-025-03720-1)
Supplement: Supplementary file 1 — (DOCX 20.9 KB) [file 10815_2025_3720_MOESM1_ESM.docx]

**Supplemental Table 1.** Summary of cohort demographics
