# Supplementary material for: NMN supplementation as a strategy to improve oocyte quality: a systematic review and transcriptomic analysis
Source: J Assist Reprod Genet. 2025 Oct 29;43(1):51–65. doi: 10.1007/s10815-025-03720-1 (PMC12831783; doi:10.1007/s10815-025-03720-1)
Supplement: Supplementary file 2 — (DOCX 753 KB) [file 10815_2025_3720_MOESM2_ESM.docx]

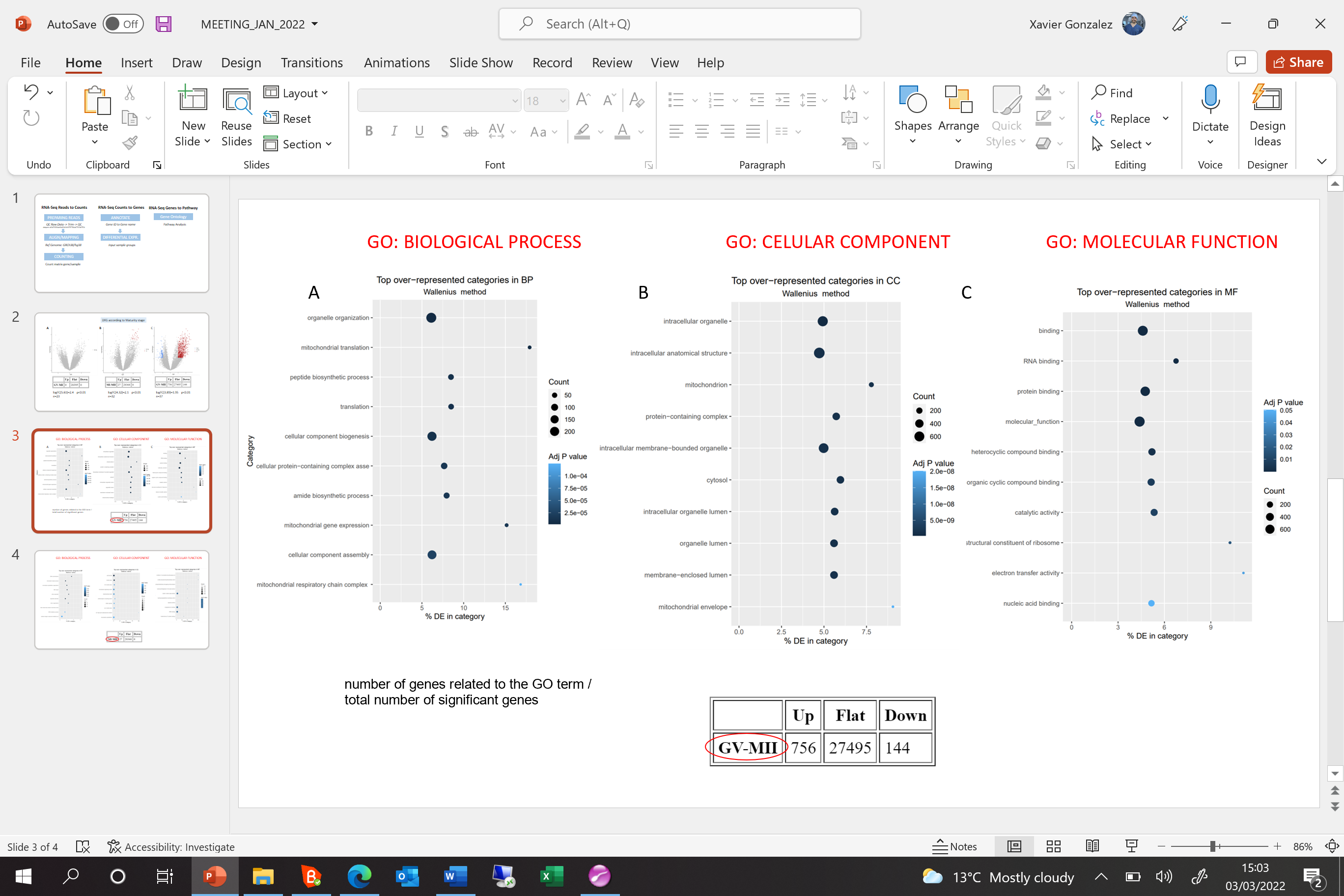


**Supplemental Figure 1.** Plots represent the top 10 over-represented GO terms considering (A) biological process, (B) cellular component and (C) molecular function. X axis represents differential expression percentage in each category, where zero represents no differential expression.
